# Differential Regulation of Angiogenesis, Lymphangiogenesis, and Neural Tissue in Normal and Inflamed Dental Pulp: Immunohistochemical Analysis

**DOI:** 10.3390/diagnostics15141819

**Published:** 2025-07-19

**Authors:** Nooruldeen Ammar Alani, Bashar Hamid Abdullah

**Affiliations:** Department of Oral Diagnostic Sciences, College of Dentistry, Baghdad University, Baghdad 10071, Iraq; alaninooraldeen74@gmail.com

**Keywords:** dental pulpitis, lymphangiogenesis, angiogenesis, pulp inflammation, D2-40, vascular imbalance

## Abstract

**Background/Objectives:** Pulp inflammation impairs healing, yet the underlying vascular and neural mechanisms remain poorly understood. This study investigated the differential regulation of lymphatic vessels, blood vessels, and neural tissue in pulpitis to elucidate healing limitations in inflamed dental pulp. **Methods:** This study evaluated 38 pulp samples (14 symptomatic irreversible pulpitis, 13 asymptomatic irreversible pulpitis, and 11 healthy controls) via immunohistochemistry, using D2-40 to identify lymphatic vessels, CD31 to mark blood vessels, and PGP9.5 to detect neural tissue. Vessel counts and neural tissue scoring were performed by blinded examiners and analyzed using appropriate statistical tests. **Results:** Dental pulp with symptomatic irreversible pulpitis exhibited significantly increased blood vessel density (50.3 vs. 39.2 in asymptomatic irreversible pulpitis and 25.8 in controls, *p* = 0.001, Cohen’s d = 1.82), while lymphatic vessel density remained unchanged across all groups (*p* ≥ 0.05), indicating impaired lymphangiogenesis despite inflammation. Neural tissue density was consistent across conditions, with a significant negative correlation between PGP9.5 expression and age (r = −0.5, *p* = 0.001). CD31 and D2-40 expression showed a positive correlation (r = 0.389, *p* = 0.016), suggesting coordinated vascular development. **Conclusions:** Our findings reveal a critical imbalance between enhanced angiogenesis and impaired lymphangiogenesis during pulpitis, potentially explaining the compromised healing capacity of inflamed dental pulp. This vascular dysregulation, combined with persistent neural tissue density, creates an environment in which inflammatory exudates accumulate with limited clearance. These insights indicate a need for new therapeutic strategies aimed at enhancing lymphangiogenesis to improve endodontic outcomes.

## 1. Introduction

Dental pulp is vascularized and innervated connective tissue located within the pulp chamber [1]. Injury due to dental caries or trauma induces an inflammatory response that depends on the balance between inflammatory processes and regenerative capability [2,3,4,5]. Managing inflammation and removing extravasated fluids is essential to maintaining pulp functionality, highlighting the critical roles of lymphatic vessels, blood vessels, and neural tissue [6,7].

The presence of lymphatic vessels in human dental pulp remains controversial. Some studies have identified lymphatic vessels in healthy dental pulp [8,9,10,11,12], while others report their absence [13,14,15]. Lymphatic vessels are vital for maintaining tissue homeostasis through the recirculation and absorption of interstitial fluid and provide a defense against bacterial infections under pathological conditions [6].

Upon activation during inflammation, sensory nerve fibers not only convey nociceptive signals but also induce neurogenic inflammation through neuropeptide release. These biomolecules trigger vasodilation, enhance vascular permeability, and facilitate immune cell recruitment and activation [16,17]. Additionally, pulp inflammation triggers the secretion of nerve growth factor (NGF), which plays a pivotal role in neuronal development, maintenance, and repair [18].

Immunohistochemistry enables the reliable identification of vascular and neural elements in dental pulp. The D2-40 monoclonal antibody specifically targets lymphatic endothelial cells [19], CD31 is expressed by both blood and lymphatic vessel endothelium [20], and PGP9.5 specifically identifies neural elements [21]. Despite the availability of these tools, the differential response of lymphatic vessels, blood vessels, and neural tissue to pulp inflammation has not been comprehensively characterized in a single study.

This study aimed to assess the presence and allocation of lymphatic vessels in normal and inflamed dental pulp. We aimed to determine whether pulp inflammation induces lymphangiogenesis, evaluate changes in blood vessel density in symptomatic and asymptomatic irreversible pulpitis, and examine alterations in neural tissue characteristics during pulp inflammation. By investigating these parameters simultaneously, we aimed to provide a comprehensive understanding of the neurovascular dynamics during pulpitis and identify potential targets for enhancing pulp healing and regeneration.

## 2. Materials and Methods

### 2.1. Statistical Reliability

Inter-examiner reliability for vessel counting demonstrated excellent agreement with an ICC of 0.92, indicating robust assessment methodology. This high level of agreement between independent examiners confirms the reliability of the vessel quantification data presented in this study.

### 2.2. Ethical Consideration

The study protocol was reviewed and approved by the Ethics Committee of Baghdad University College of Dentistry (protocol code 878). Written informed consent was obtained from all participants or their legal guardians prior to tissue collection and examination, in accordance with the Declaration of Helsinki.

### 2.3. Demographic Characteristics

Thirty-eight pulps were extirpated using a barbed broach. Fourteen pulps were diagnosed with symptomatic irreversible pulpitis, and thirteen pulp samples were histologically diagnosed with asymptomatic irreversible pulpitis. The extirpation was performed for endodontic reasons. The remaining 11 pulp tissues were extirpated immediately after the extraction of normal teeth for orthodontic reasons or because of an impacted third molar [22,23]. The extirpation approach was specifically chosen over decalcification methods to preserve tissue antigenicity and avoid the false-positive staining of D2-40 by lymphatics in the periodontal ligaments. All samples were submitted for histopathological evaluation using H&E staining for accurate diagnosis. Microscopic evaluation was performed with a leica DM750, and the image was captured using a leica ICC50 E microscope camera. The ethics committee of the Baghdad University College of Dentistry approved this study (protocol code: 878). Informed consent was obtained from all participants prior to tissue collection and examination.

### 2.4. Diagnostic Criteria

Diagnosis of the pulpal state was based on the clinical and histopathological criteria recommended by the American Association of Endodontists [24]. For symptomatic irreversible pulpitis, the clinical diagnosis required spontaneous, severe pain or lingering pain to thermal stimuli. Histologically, these cases were confirmed by the predominance of a neutrophilic infiltrate and vascular dilation.

For asymptomatic irreversible pulpitis, the clinical diagnosis was based on the presence of deep carious lesions requiring endodontic treatment but with minimal or no spontaneous pain. Histologically, these cases were confirmed by the presence of a chronic inflammatory cell infiltrate (lymphocytes, plasma cells, and macrophages), fibrosis, and often dystrophic calcifications.

Normal pulp samples exhibited no clinical symptoms and demonstrated an absence of inflammatory cells with maintained normal tissue architecture upon histological examination.

### 2.5. Immunohistochemistry

All specimens were fixed in 10% buffered formalin for 24 h. Following fixation, the specimens were embedded in paraffin wax, and 4 μm thick sections were prepared for immunohistochemical examination. The procedure included dewaxing and rehydration, followed by antigen retrieval using a heat-mediated method in a water bath at a pH of 6 for CD31 and Podoplanin and a pH of 9 for PGP9.5 (Pathinsitu). After washing with immune buffer for 5 min, hydrogen peroxide blocking was performed, followed by two 5 min washes and protein blocking. The tissue was washed twice for 5 min (Abcam) before the primary antibodies were applied.

Three primary mouse monoclonal antibodies specific for human antigens were used: anti-CD31 (0.5 μg/mL; positive control: human tonsil), anti-PGP9.5 (2 μg/mL; positive control: human brain), and anti-podoplanin (1/10,000 dilution; positive control: human placenta). These primary antibodies were incubated overnight and rinsed 4 times in a buffer, followed by the application of biotinylated goat anti-polyvalent (10 min incubation; 4 washes). Streptavidin peroxidase was applied for 10 min, followed by 4 buffer rinses. DAB chromogen, diluted in a DAB substrate according to manufacturer specifications, was applied for 7 min, followed by 4 rinses. Harris hematoxylin was used as a counterstain before cover slipping.

### 2.6. Quantification and Assessment

To count vessels, five high-power fields (40× magnification) were systematically selected from each sample: three fields from the coronal pulp and two from the radicular pulp. All positively stained vessels in each field were counted, and the mean vessel density was calculated for each sample. To ensure reliability, all counting was performed by two independent examiners who were blinded to the sample diagnoses, with any discrepancies resolved by consensus.

For nerve tissue assessment, PGP9.5 immunoreactivity was scored on a scale from 0 to 4, where 0 represented negative staining, 1 represented less than 10% positive staining, 2 represented 10–25% positive staining, 3 represented 25–50% positive staining, and 4 represented more than 50% positive staining.

### 2.7. Statistical Analysis

A data analysis was performed using Statistical Package for Social Sciences (SPSS), version 26. The results are presented as means, standard deviations, 95% confidence intervals, and ranges. Categorical data are presented as frequencies and percentages. The Shapiro–Wilk test was used to test the normality of the data distribution. An analysis of variance (ANOVA) (two-tailed) was used for normally distributed data, and the Kruskal–Wallis test was used for abnormally distributed data to compare the biomarkers between study groups. For pairwise comparisons, Tukey’s post hoc test was applied following the ANOVA, and Dunn’s test with the Bonferroni correction was used following the Kruskal–Wallis test. Pearson’s correlation coefficient assessed relationships between continuous variables. A post hoc power analysis was conducted using G*Power 3.1 to determine the statistical power of our sample size. For the primary outcome (differences in CD31 expression between groups), with a total sample size of 38, an alpha of 0.05, and the large effect size observed (f = 0.91, equivalent to Cohen’s d = 1.82). Inter-examiner reliability for vessel counting was assessed using an intraclass correlation coefficient (ICC). A *p* value less than 0.05 was considered statistically significant.

## 3. Results

### 3.1. Demographic Characteristics:

A total of 38 pulp tissues were analyzed in this study, comprising 14 with symptomatic irreversible pulpitis, 13 with asymptomatic irreversible pulpitis, and 11 normal pulp samples. The gender distribution showed no significant differences between groups, with 42.9% male (n = 6) and 57.1% female (n = 8) in the symptomatic irreversible pulpitis group, 46.2% male (n = 6) and 53.8% female (n = 7) in the asymptomatic irreversible pulpitis group, and 36.4% male (n = 4) and 63.6% female (n = 7) in the normal pulp group. The mean age of the individuals in this study was 24.23 ± 6.8 years. No significant differences in age were observed between the study groups (*p* = 0.78).

### 3.2. Immunohistochemical Findings

**Figure 1:**
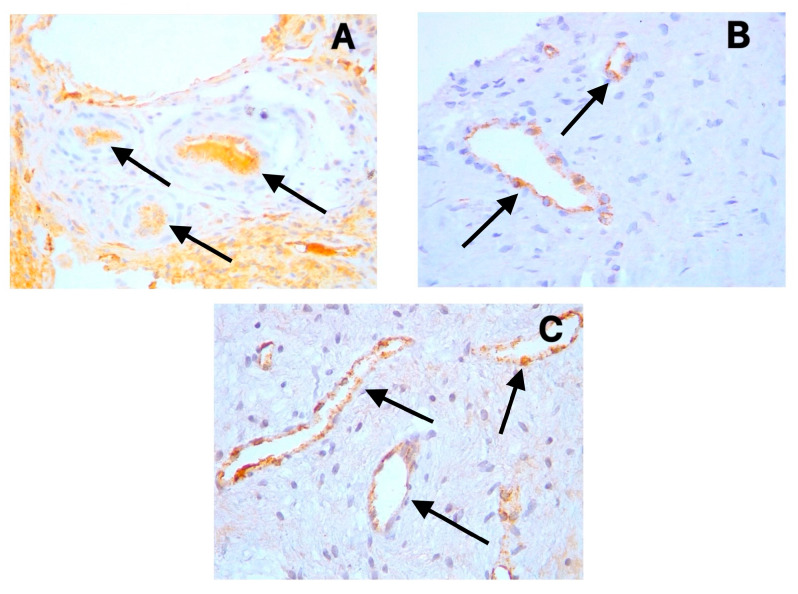
Positive cytoplasmic expression of D2-40 in endothelial cells of lymphatic vessels (black arrows) in (**A**) symptomatic irreversible pulpitis, (**B**) asymptomatic irreversible pulpitis, and (**C**) normal pulp tissue.

**Figure 2:**
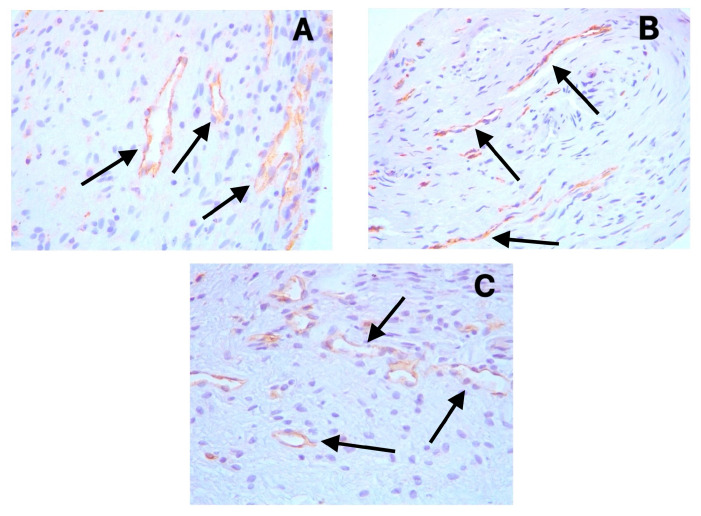
Distinct cytoplasmic expression of CD31 in endothelial cells of both blood and lymphatic vessels (black arrows) in (**A**) symptomatic irreversible pulpitis, (**B**) asymptomatic irreversible pulpitis, and (**C**) normal pulp tissue.

**Figure 3:**
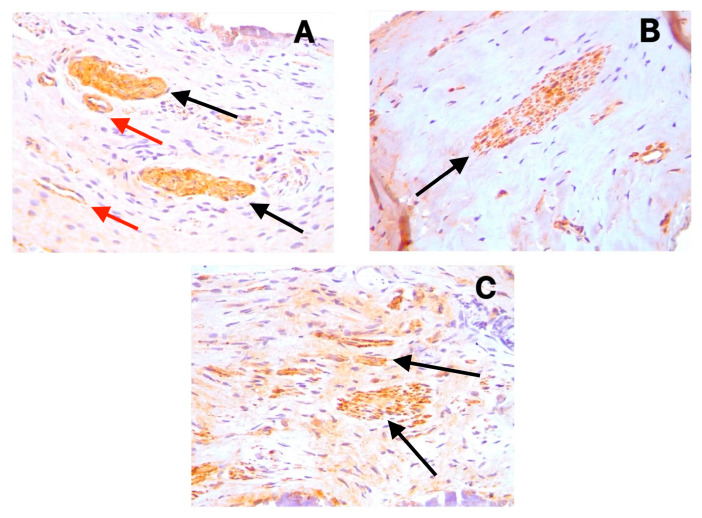
Cytoplasmic expression of PGP9.5 in neuronal cells (black arrows) and pericytes surrounding blood vessel walls (red arrows) in (**A**) symptomatic irreversible pulpitis, (**B**) asymptomatic irreversible pulpitis, and (**C**) normal pulp tissue.

The positive cytoplasmic expression of D2-40 (Podoplanin) was observed in the endothelial cells of lymphatic vessels across all sample types (Figure 1A–C). These lymphatic vessels were primarily distributed in the coronal pulp, with fewer observed in the radicular region. CD31 showed distinct cytoplasmic staining in the endothelial cells of both blood and lymphatic vessels (Figure 2A–C), with notably increased expression in the symptomatic irreversible pulpitis samples. PGP9.5 exhibited cytoplasmic expression in neuronal cells and pericytes surrounding blood vessel walls, with a relatively uniform distribution from coronal to radicular pulp across all groups (Figure 3A–C).

**Figure 4:**
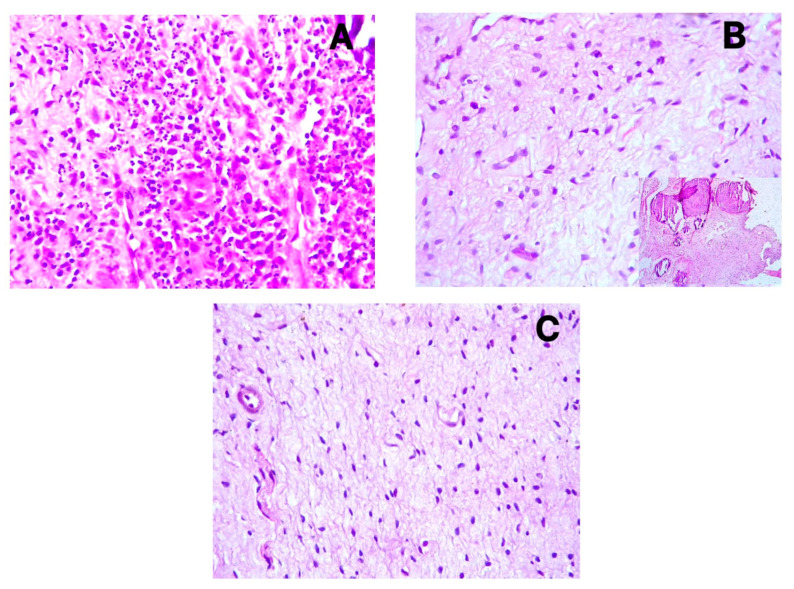
H&E staining for histopathological diagnosis reveals (**A**) inflammatory cell infiltrate predominantly comprising neutrophils in cases of symptomatic irreversible pulpitis; (**B**) inflammatory cell infiltrate characterized by lymphocytes, accompanied by fibrosis and calcification (a notable insight) in asymptomatic irreversible pulpitis; and (**C**) an absence of inflammatory cells and inflammatory features in normal pulp.

The distribution of blood and lymphatic vessels was more pronounced in the coronal pulp than in the radicular pulp. However, during inflammation, particularly in symptomatic irreversible pulpitis, increased angiogenesis was observed even in the radicular pulp regions. H&E staining confirmed the histopathological diagnoses of symptomatic irreversible pulpitis (Figure 4A), asymptomatic irreversible pulpitis (Figure 4B), and healthy pulp (Figure 4C), validating the clinical classification of the samples.

### 3.3. Vessel and Nerve Density Analysis

The symptomatic irreversible pulpitis samples demonstrated a significant increase in blood vessel density, as measured by CD31 immunoexpression (mean 50.3, 95% CI 43.1–57.5), compared to the asymptomatic irreversible pulpitis samples (mean 39.2, 95% CI 32.7–45.7) and control samples (mean 25.8, 95% CI 19.4–32.2) (*p* = 0.001) (Table 1). The effect size for this difference (Cohen’s d = 1.82 between symptomatic irreversible pulpitis and control samples) indicates a large and clinically significant effect. In contrast, no significant differences were observed in lymphatic vessel density (D2-40 expression) or nerve tissue density (PGP9.5 expression) between the study groups (*p* ≥ 0.05).

Post hoc pairwise comparisons revealed that CD31+ vessel density was significantly higher in the symptomatic irreversible pulpitis samples compared to both the asymptomatic irreversible pulpitis (50.3 vs. 39.2, *p* = 0.031) and control (50.3 vs. 25.8, *p* = 0.001) samples. However, no significant difference was observed between the asymptomatic irreversible pulpitis and control samples (39.2 vs. 25.8, *p* = 0.386). This suggests that increased angiogenesis is primarily associated with symptomatic irreversible pulpitis rather than asymptomatic irreversible pulpitis in dental pulp.

### 3.4. Correlation Analysis

A significant positive correlation was observed between CD31 and D2-40 expression (r = 0.389, *p* = 0.016), suggesting coordinated development between blood and lymphatic vessels during pulpitis. However, no significant correlations were detected between PGP9.5 expression and either CD31 or D2-40 (*p* ≥ 0.05) (Table 2), indicating that neural tissue regulation may follow pathways independent of vascular changes.

**Figure 5:**
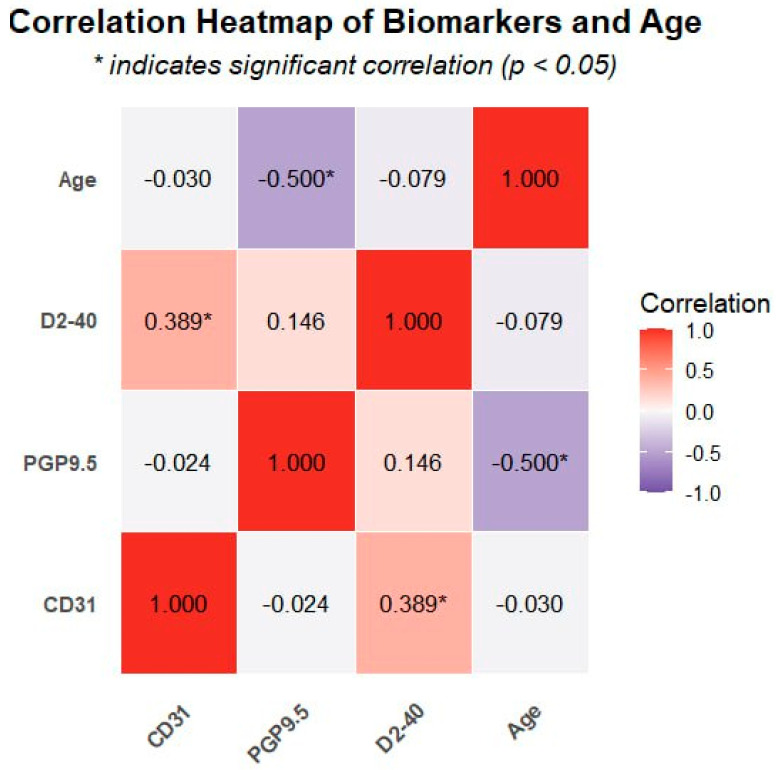
Heatmap presenting correlations between all biomarkers and age. The Pearson correlation coefficients (r) between age and the expression levels of different biomarkers (CD31, PGP9.5, D2-40) are represented by the values in the cells. Red denotes a positive correlation (r > 0), blue/purple denotes a negative correlation (r < 0), and white denotes no correlation (r ≈ 0). * Denotes a statistically significant correlation at *p* < 0.05.

Notably, a significant negative correlation between PGP9.5 expression and age (r = −0.5, *p* = 0.001) was found, indicating a decrease in neural tissue density with advancing age. In contrast, neither CD31 nor D2-40 expression showed a significant correlation with age (*p* ≥ 0.05), suggesting that vascular components may be less affected by aging compared to neural elements. The correlation patterns between all biomarkers and age are visually represented in the heatmap (Figure 5), where the intensity of color indicates the strength of correlation and asterisks mark statistically significant relationships.

## 4. Discussion

### 4.1. Lymphatic Vessels in Dental Pulp

The omnipresence of lymphatic vessels in human dental pulp remains a contentious issue, particularly regarding whether inflammation in the dental pulp induces lymphangiogenesis. Our study provides important clarification on this controversial topic by demonstrating the presence of lymphatic vessels in all pulp samples examined, including normal pulp, using the highly specific D2-40 marker. This finding is significant because it resolves a fundamental question in pulp biology and contradicts several previous studies that reported the absence of lymphatics in dental pulp.

Numerous studies employing immunohistochemical techniques have affirmed the presence of lymphatic vessels in the dental pulp across various species [8,9,10,11,12]. Conversely, multiple authors have reported the absence of such vessels in the dental pulp of differing species [13,14,15]. Gerli et al. assert that true lymphatic vessels are not typically present in human dental pulp but may emerge following inflammatory responses. Under physiological conditions, interstitial fluid that is not reabsorbed on the venular side would flow through ‘non-endothelialized interstitial channels’ [13]. Martin et al. concluded that lymphatic vessels are absent in the dental pulp of canines, suggesting fundamental similarities in the endodontic biology of dogs and humans, which aligns with findings by Gerli et al. [14].

Our study utilized D2-40, a marker with high specificity for lymphatic endothelium [25], demonstrating that lymphatic vessels were present in all examined samples, including normal pulp samples. This finding contradicts studies reporting the absence of lymphatics in dental pulp but aligns with others that have identified these vessels [8,9,10,11,12]. Additionally, the endothelium of these vessels expresses CD31, confirming the existence of an endothelial lining, given that CD31 serves as a pan-endothelial marker for both blood and lymphatic vessels [20].

Our data indicate a moderate positive correlation between the expression of CD31 and that of D2-40 (r = 0.389), suggesting some degree of coordinated development between blood and lymphatic vessels. However, the density of lymphatic vessels in samples with symptomatic and asymptomatic irreversible pulpitis does not significantly differ from that of control samples. This finding is particularly noteworthy as it suggests that, unlike angiogenesis, lymphangiogenesis appears to be inhibited during pulp inflammation, contradicting some studies that reported substantial lymphangiogenesis in inflamed pulp.

This impairment of lymphangiogenesis may be attributable to elevated levels of nitric oxide (NO) produced during the inflammatory response. Nitric oxide interacts with superoxide (O2−) to yield toxic concentrations of peroxynitrite (OONO−) [26]. According to Singla B. et al., low levels of reactive oxygen species (ROS) are necessary for lymphangiogenesis, whereas excessive ROS production can inhibit lymphangiogenesis and impair lymphatic drainage [27]. This molecular mechanism represents a potential therapeutic target for enhancing pulp healing by modulating the inflammatory microenvironment to promote lymphangiogenesis.

The inability to form new lymphatic vessels likely adversely influences the healing and regenerative capacity of the dental pulp, hindering the reabsorption of extravasated fluid and promoting its accumulation. This state predisposes the pulp to circulatory failure and potential necrosis. These findings have significant clinical implications, suggesting that therapeutic approaches aimed at enhancing lymphangiogenesis might improve pulp healing outcomes in pulpitis.

### 4.2. Blood Vessel Changes in Pulpitis

Our results demonstrate a striking difference in angiogenic response between symptomatic and asymptomatic irreversible pulpitis. Although the results indicate an increased presence of blood vessels in pulpitis during both symptomatic and asymptomatic irreversible pulpitis, this increase is notably more pronounced under symptomatic irreversible pulpitis. The mean vessel density in symptomatic irreversible pulpitis samples (50.3) was nearly twice that of normal pulp samples (25.8), representing a large effect size (Cohen’s d = 1.82). In contrast, the changes observed in asymptomatic irreversible pulpitis cases appear insignificant when compared to the normal group.

The significant angiogenesis noted in the symptomatic irreversible pulpitis samples can be attributed to hypoxic conditions. Hypoxia serves as a potent modulator of repair signals across various tissues, including dental pulp, as injuries—whether resulting from trauma or microbe-induced inflammation—often damage vascular structures, resulting in diminished oxygen supply to the tissue. Dental pulp cells subjected to hypoxia act as the driving force behind new blood vessel formation. Furthermore, nitric oxide and other cytokines released by immune cells, fibroblasts, and odontoblasts propel angiogenesis [28].

These findings highlight the dynamic nature of the vascular response during different stages of pulpitis and suggest that therapeutic strategies might need to be tailored differently for symptomatic versus asymptomatic irreversible pulpitis. The robust angiogenic response in symptomatic irreversible pulpitis may represent an attempt at tissue repair that could potentially be harnessed therapeutically.

### 4.3. Neural Tissue Response to Inflammation

Beyond pain signal transmission, sensory nerve fibers can induce neurogenic inflammation through the secretion of neuropeptides [29], particularly calcitonin gene-related peptide (CGRP) and substance P (SP), in response to inflammatory stimuli. This process leads to an increase in vascular permeability, vasodilation, and angiogenesis, coupled with the recruitment and activation of immune cells [30,31].

The findings of this study reveal no significant variation in the density of nerve fibers (as indicated by PGP9.5 expression) in symptomatic and asymptomatic irreversible pulpitis compared to normal pulp. This stability in nerve density is notable since inflammation often damages neural tissues. We hypothesize that dynamic regulation by nerve growth factor (NGF) facilitates neuronal differentiation [29], enhances nerve tissue density, and compensates for damaged nerve fibers. This suggests that neuropeptide secretion persists during inflammation, maintaining neurogenic inflammation in the dental pulp and engendering continuous pro-inflammatory mediator production.

Additionally, we observed a significant negative correlation between PGP9.5 expression and age (r = −0.5, *p* = 0.001), indicating a decrease in neural tissue density with advancing age. This finding is consistent with age-related neurodegeneration processes reported in other tissues [32]. This age-related decline in neural elements might have implications for diagnosis and treatment outcomes in older patients with pulpitis, suggesting that age should be considered as a factor in treatment planning.

### 4.4. Clinical Implications and Limitations

The vascular and neural dynamics observed in our study offer important clinical insights. The imbalance between enhanced angiogenesis and impaired lymphangiogenesis likely creates an environment in which inflammatory exudates accumulate, increasing intrapulpal pressure and pain. This understanding could inform targeted pain management approaches for patients with pulpitis. The maintenance of neural tissue density during inflammation validates the continued use of pulp sensitivity testing throughout the disease process, though clinicians should consider potentially reduced test reliability in older patients due to age-related neural density decline.

From a therapeutic perspective, our findings suggest that modulating the inflammatory environment to promote lymphangiogenesis while controlling excessive angiogenesis could improve pulp healing outcomes. Regenerative endodontic procedures might benefit from incorporating lymphangiogenic factors to enhance fluid drainage and reduce inflammatory pressure.

Our study has several limitations. The relatively small sample size may limit statistical power for detecting subtle differences between groups. Furthermore, the samples were collected from various tooth types, which could introduce variability, and this was not standardized in our analysis. Our immunohistochemical approach identified structures but could not assess functional lymphatic or vascular flow. The cross-sectional design prevented the observation of temporal changes during disease progression, and we did not examine molecular mediators that could provide mechanistic insights into the differential vascular regulation observed.

### 4.5. Future Directions

Future research should explore the molecular mechanisms inhibiting lymphangiogenesis during pulp inflammation, particularly the ROS and nitric oxide pathways. Investigating whether lymphangiogenic therapies improve pulp healing outcomes would be valuable for developing targeted treatments. Additional studies examining the relationship between NGF expression and nerve fiber maintenance during inflammation could identify neuroprotective strategies for pulp preservation. Longitudinal studies tracking vascular and neural changes during pulpitis progression would provide temporal insights, while a more extensive investigation of age-related changes in pulp vasculature and innervation could inform age-appropriate treatment protocols.

## 5. Conclusions

This study demonstrates that lymphatic vessels are present in all dental pulp samples, yet lymphangiogenesis remains impaired during pulpitis despite a significant enhancement of angiogenesis in symptomatic irreversible pulpitis. This vascular imbalance—characterized by increased blood vessel formation without corresponding lymphatic development—likely contributes to inadequate clearance of inflammatory mediators and compromised healing in pulpitis. Our findings also reveal that neural tissue density remains stable during inflammation despite age-related decreases, suggesting persistent neurogenic inflammation throughout the disease process. These insights into the differential regulation of vascular and neural elements during pulpitis provide a foundation for the development of targeted therapeutic approaches that promote lymphangiogenesis and improve pulp healing outcomes.

## Figures and Tables

**Table 1:** Biomarker expression across study groups.

Biomarker	Symptomatic Irreversible Pulpitis	Asymptomatic Irreversible Pulpitis	Control	*p*-Value
CD31 *	50.3 (25.5–88.2)	39.2 (26.2–49.8)	25.8 (17.8–49.0)	0.001
PGP9.5 *	4.0 (2.0–4.0)	3.0 (1.0–4.0)	4.0 (3.0–4.0)	0.161
D2-40 †	28.35 ± 10.6	24.72 ± 9.0	22.72 ± 6.8	0.299

* Values are presented as median (range). † Values are presented as mean ± standard deviation.

**Table 2:** Correlation matrix between biomarkers and age.

Variable	CD31	PGP9.5	D2-40	Age
CD31	1.0	−0.024	0.389 *	−0.03
PGP9.5	−0.024	1.0	0.146	−0.500 **
D2-40	0.389 *	0.146	1.0	−0.079
Age	−0.03	−0.500 **	−0.079	1.0

* Correlation is significant at *p* < 0.05 level. ** Correlation is significant at *p* < 0.01 level.

## Data Availability

The raw data collected by the researchers for statistical analysis have been stored and can be provided upon request.
